# Bamboo Shoot Shell Powder-Regulated High-Moisture Extruded Soy Protein Isolate: Improving Fibrous Structure and Physicochemical Properties

**DOI:** 10.3390/foods15213919

**Published:** 2026-11-02

**Authors:** Lihua Wen, Jingfeng Qin, Mengjing Zhu, Yuqin Huang, Yan Gao, Zebin Guo, Shuchun Xu, Dongtensheng Yu, Haoran Chen, Baodong Zheng

**Affiliations:** 1 College of Food Science, Fujian Agriculture and Forestry University, Fuzhou 350002, China; wlhfst@163.com (L.W.); jf11451@163.com (J.Q.); zmjfst1@163.com (M.Z.); 18850157350@163.com (Y.H.); 15245483510@163.com (Y.G.); gzb8607@163.com (Z.G.); 2 Fujian Provincial Key Laboratory of Quality Science and Processing Technology in Special Starch, Fujian Agriculture and Forestry University, Fuzhou 350002, China; 3 Fujian Lianhua Qigong Bamboo Shoot Industry Co., Ltd., Jianou 353100, China; edit2k@gmail.com (S.X.); dongtensheng@163.com (D.Y.)

**Keywords:** bamboo shoot shells, multiscale structure, high-moisture extrusion, soy protein isolate

## Abstract

Textured vegetable proteins produced by high-moisture extrusion often exhibit limited controllability of fibrous structure, restricting their utilization in plant-based meat analogues. In this work, bamboo shoot shell powder (BSSP) was incorporated during the high-moisture extrusion of soy protein isolate (SPI) to modulate the multiscale structure and functionality of the resulting extrudates. During high-moisture extrusion, the incorporation of 5–10% BSSP promoted the evolution of extrudate microstructures into channel-like pores aligned along the extrusion direction. Moderate BSSP addition (~15%) increased β-sheet and β-turn contents while decreasing α-helix and random coil fractions. These conformational transitions, linked to enhanced hydrogen bonding and weakened hydrophobic interactions, generated extrudates with improved thermal stability. Moreover, the addition of 15~20% BSSP increased the hardness and chewiness of the extrudates, as revealed by texture profile analysis. BSSP incorporation also enhanced the freeze–thaw stability of the extrudates while reducing bitterness. These findings suggested that BSSP acted as a functional structural regulator, offering an approach for tailoring plant-based meat analogues with improved microstructural organization and textural properties.

## 1. Introduction

The worldwide demand for meat production has shown a consistent increase in recent decades, largely attributed to expanding populations, rapid urbanization, and increasing consumer purchasing power [1]. This expansion has placed substantial resource and environmental pressures on conventional animal-derived protein supply. Under these constraints, developing meat analogues from plant proteins has emerged as a promising route toward a more sustainable protein system. Textured vegetable proteins (TVPs) are the primary structural basis of many plant-based meat products, and their moisture level critically influences the resulting morphology and eating quality. Low-moisture TVPs (10–40% moisture) typically exhibit a loose, sponge-like porous structure that requires rehydration before use, thereby limiting their application primarily to meat additives [2]. By contrast, high-moisture TVPs (40–80% moisture) form a dense fibrous network during extrusion, enhancing elasticity and juiciness while generating a hierarchical architecture that more closely mimics native muscle tissue [3].

High-moisture extrusion (HME) is a continuous thermal–mechanical processing approach that combines thermal treatment, shear-induced, and directional structuring [4]. By precisely controlling screw temperature, moisture content, and die cooling conditions, this technology facilitates protein realignment and fibrous structure formation under high-moisture conditions [5]. Concurrently, the combined effects of elevated temperature and intense shear drive proteins to transition from an initial solid state into a viscoelastic molten phase through denaturation and molecular unfolding [6]. As the molten matrix flows through the cooling die, the temperature rapidly drops into the phase-separation regime, inducing spatial segregation of the continuous water phase and protein-rich phases. These structures align along the flow direction and are subsequently fixed upon solidification, resulting in an anisotropic and lamellar fibrous network. Nonetheless, high-moisture extrudates always exhibit insufficient structural stability of the fibrous architecture and may retain undesirable sensory attributes. These limitations primarily stem from the difficulty in precisely controlling intermolecular protein interactions and aggregation pathways during extrusion.

Incorporating dietary fibers into protein systems has proven to be a promising approach for tailoring the structural and functional properties of extrudates [7]. Non-starch polysaccharides could modulate protein unfolding and crosslinking during extrusion by affecting melt viscoelasticity, water distribution, and phase separation [8]. Insoluble dietary fibers act as stress-transmitting scaffolds to promote protein alignment [9], while soluble components stabilize networks via hydrogen bonding [10]. Bamboo shoots are a dietary fiber–rich resource characterized by a porous microstructure and high water-holding capacity. Beyond the edible portion, large quantities of by-products (e.g., bamboo shoot shells and basal residues) are generated during industrial processing, which are often discarded and may impose environmental burdens if not properly utilized. Therefore, valorizing these by-products as functional ingredients represents an effective strategy to enhance the sustainable utilization of plant resources. Notably, the dietary fibers derived from bamboo shoot shells exhibit greater structural rigidity and thermal stability than cereal-derived counterparts and have been reported to modulate protein gelation behavior [11]. Despite these promising functional attributes, studies investigating the incorporation of bamboo shoot shells into plant-based meat analogs remain unexplored. The underlying mechanisms by which dietary fibers regulate protein structural transitions and aggregation pathways during high-moisture extrusion remain unclear.

In this work, the multiscale regulatory role of bamboo shoot shells during the high-moisture extrusion of soy protein isolate is investigated. By incorporating bamboo shoot shell powder at varying levels, we evaluated their impact on systemic processing responses, intermolecular interactions and the freeze–thaw stability. These findings provided insight into the structure–function relationships in fiber-assisted protein texturization, offering a theoretical basis for optimizing the textural properties of plant-based meat analogues.

## 2. Materials and Methods

### 2.1. Materials

Bamboo shoot shells (Moso bamboo) were purchased from Fujian Lianhua Qigong Food Co., Ltd. (Jianou, China). According to compositional analysis, the bamboo shoot shells consisted of (*w*/*w*): insoluble dietary fiber (38.7% ± 0.5%), soluble dietary fiber (2.7% ± 0.1%), moisture (5.5% ± 0.3%), protein (15.0% ± 0.5%), ash (3.94% ± 0.2%), and fat (2.9% ± 0.1%). Soy protein isolate (SPI), containing 82.8 wt% protein, was supplied by Shandong Yuwang Ecological Food Industry Co., Ltd. (Yucheng, China). Thioflavin T (ThT, purity ≥ 98%) and the protein concentration assay kit were obtained from Solarbio (Beijing, China). All other reagents used in this study were of analytical grade.

### 2.2. Preparation of the SPI-BSSP Blends

Fresh bamboo shoot shells were washed and dried at 45 °C for 24 h, milled, and passed through an 80-mesh sieve to obtain bamboo shoot shell powder (BSSP). The prepared powder was stored in a desiccator until further use. The BSSP was homogeneously blended with soy protein isolate (SPI) at mass ratios of 100/0, 95/5, 90/10, 85/15, 80/20, and 75/25, and the resulting mixtures were used as feed materials for high-moisture extrusion (HME).

### 2.3. High-Moisture Extrusion Trials and Sample Collection

The HME experiments were performed on a parallel co-rotating twin-screw extruder (Process 16 Hygienic, ThermoFisher Scientific, Waltham, MA, USA) with a screw diameter of 16 mm and an L/D ratio of 40. The extruder comprised eight independently controlled barrel segments and seven top feeding ports. To avoid material build-up in the feeding zone, the first barrel segment was continuously cooled, whereas the remaining segments were equipped with electric heating units with independent temperature control. At the barrel outlet, a liquid-cooled slit die (188 mm in length) was installed, with its temperature accurately regulated by a thermostatic cooling system.

Prior to extrusion, the prepared mixtures were conveyed into the first barrel inlet via a volumetric twin-screw feeder (DDSR20, Thermo Fisher Scientific, Waltham, MA, USA). The solid feeding rate (g/min) and the initial moisture content (%, *w*/*w*) were determined gravimetrically using an electronic balance and a moisture analyzer (MFA-120A, Lianou World Instrument Co., Ltd., Suzhou, China), respectively. Additionally, deionized water was delivered into the third barrel inlet by a digital peristaltic pump (BT100LC, Chuangrui Precision Pump Industry Co., Ltd., Baoding, China). The water feed rate (FRw) was adjusted according to the initial moisture content and solid feeding rate to maintain the final moisture content of the extrudates at approximately 65%. During high-moisture extrusion, the required *FR_w_* (g/min) was calculated based on the following equation:

$${\mathrm{FR}}_{w\;}=\;\frac{M_{t}{-M}_{s}}{{1-M}_{t}}\;\times \;{\mathrm{FR}}_{s}$$

where *FR_S_*, *M_t_* and *M_S_* represent the solid feeding rate (g/min), the target moisture content (65%), and the initial moisture content of the protein matrix, respectively.

**Figure 1:**
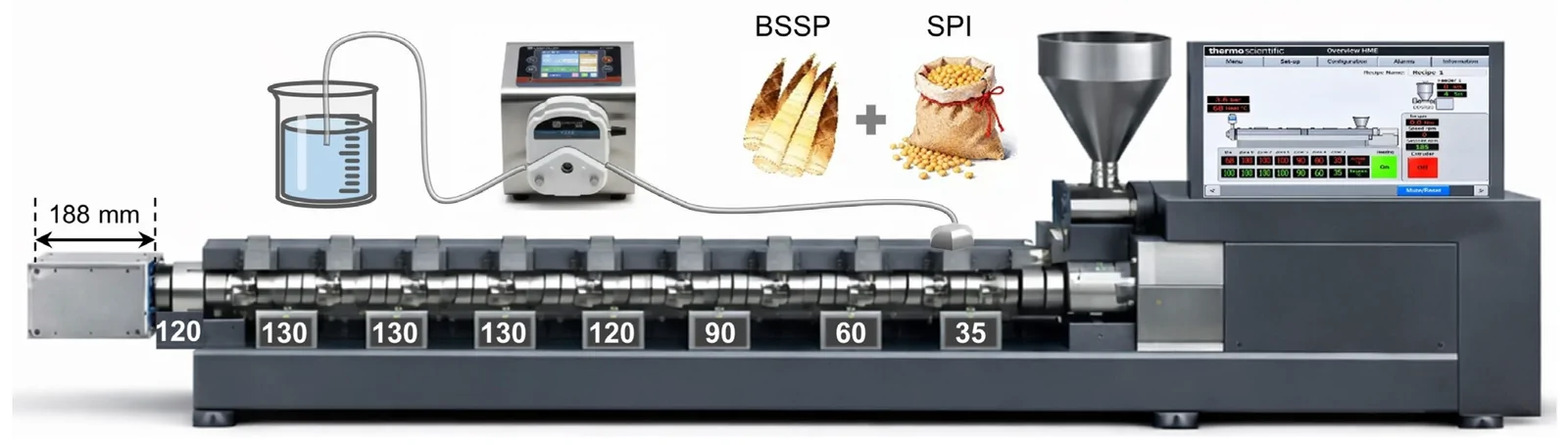
Schematic diagrams of the high-moisture extrusion system.

Based on preliminary optimization experiments, the screw speed was fixed at 180 rpm, and the solid feed rate was maintained at 3 g/min. The temperatures of the eight heating zones were set to 35, 60, 90, 120, 130, 130, 130, and 120 °C, respectively (Figure 1). The cooling die temperature was maintained at 70 °C using circulating water, yielding extrudates with a final moisture content of approximately 65%. During transitions between formulations, extrusion was continued until the preceding material had been discharged and stable processing conditions were re-established. Samples were collected only after the extrusion process reached a steady state to minimize the influence of material transition. Meanwhile, screw torque (N·m), extrusion throughput (g/min), die pressure (bar), and melt temperature (°C) were recorded simultaneously. The extrudates were designated as SPI-BSSP5, SPI-BSSP10, SPI-BSSP15, SPI-BSSP20, and SPI-BSSP25 according to the BSSP incorporation level. The extrudate sample without BSSP was designated as SPI. Following the procedures described in Section 2.9 and Section 2.10, the extrudates were cut into specified dimensions and freeze-dried using a lyophilizer for subsequent analyses.

### 2.4. Scanning Electron Microscopy (SEM)

After thawing, the extrudates were cut along the planar direction to obtain slices of approximately 2 mm. The samples were subsequently freeze-dried and mounted onto SEM stubs, followed by sputter-coating with a thin layer of gold to enhance surface conductivity. The microstructural morphology was observed using the scanning electron microscope (GeminiSEM 300, Carl Zeiss, Oberkochen, Germany) operated at an accelerating voltage of 10.0 kV. Representative images were acquired at magnifications of 200×, 1000×, and 5000×.

### 2.5. FTIR Spectroscopy

A Nicolet iS20 spectrometer was used to perform FTIR spectroscopy for analyzing the structural characteristics of the samples (Thermo Fisher Scientific, Madison, WI, USA). Briefly, the extrudates were blended with KBr powder at a ratio of 1:100 (*w*/*w*) and finely ground under an infrared lamp, followed by compression into a transparent pellet under vacuum conditions. The pellets were immediately transferred to the FTIR spectrometer to minimize moisture interference. Spectra were recorded in the wavenumber range of 500–4000 cm^−1^ with a resolution of 4 cm^−1^, averaging 64 scans per measurement [12]. The amide I region (1600–1700 cm^−1^) was analyzed using PeakFit software (Version 4.12, Systat Software Inc., Palo Alto, CA, USA). Following baseline correction and peak deconvolution, the overlapping bands were fitted to determine the relative proportions of β-sheet, β-turn, α-helix, and random coil structures. The relative proportion of each secondary structure was calculated as the percentage of its corresponding fitted peak area to the total fitted peak area.

### 2.6. Thioflavin (ThT) Fluorescence Measurement

ThT fluorescence measurements were conducted following the method described by Xiao et al. [13]. ThT powder was dissolved in phosphate buffer (10 mmol/L, pH 7.0) to obtain a 3 mmol/L stock solution. The filtered ThT solution was subsequently diluted with the same buffer at a volume ratio of 1:50 to prepare the working solution. For fluorescence measurements, 96 μL of protein solution (1%, *w*/*w*) was mixed with 8 mL of the ThT working solution and allowed to incubate for 2 min. A reagent blank containing ThT working solution without protein was used for background fluorescence correction. The samples were subjected to synchronous fluorescence scanning using a fluorescence spectrometer (RF-6000, Shimadzu Corporation, Kyoto, Japan), with excitation and emission wavelengths in the range of 200–600 nm.

### 2.7. Measurement of Intermolecular Interaction Forces

The intermolecular bonding composition of the samples was evaluated using five chemical solvent systems, based on the approach reported by Liu et al. [14]. Based on the selective disruptive effects of different solvents on specific molecular interactions, the solvent systems were defined as follows: (A) 0.05 mol/L NaCl solution (PBS buffer, pH ≈ 7.4); (B) 0.6 mol/L NaCl solution (PBS); (C) 0.6 mol/L NaCl supplemented with 1.5 mol/L urea (PBS); (D) 0.6 mol/L NaCl supplemented with 8.0 mol/L urea (PBS); and (E) 0.6 mol/L NaCl supplemented with 8.0 mol/L urea and 0.5 mol/L β-mercaptoethanol (PBS).

The samples were dispersed in each solvent to obtain protein suspensions with a mass fraction of 2.5% (*w*/*v*). The suspensions were incubated in a thermostatic water bath (25 °C) with continuous shaking (180 rpm, 1 h) to ensure sufficient solubilization. Following centrifugation (5000× *g*, 10 min), a 100 μL aliquot of the resulting supernatant was reacted with 1.0 mL of Bradford reagent. To minimize potential interference from urea and β-mercaptoethanol, solvent-specific blanks and matrix-matched calibration curves were employed. After incubation, the absorbance was measured at 595 nm using a microplate reader (SpectraMax iD3, Molecular Devices, San Jose, CA, USA), and protein concentrations were calculated based on a standard calibration curve. Based on the differences in protein solubility among the solvent systems, the intermolecular interactions were estimated as follows: (1) ionic interactions were calculated as the protein concentration difference between solvents B and A; (2) hydrogen bonding interactions were calculated as the difference between solvents C and B; (3) hydrophobic interactions were calculated as the difference between solvents D and C; and (4) disulfide bond interactions were calculated as the difference between solvents E and D.

### 2.8. Thermogravimetric Analysis (TGA)

Approximately 5 mg of samples was loaded into an alumina crucible and analyzed using a thermogravimetric analyzer (TGA 8000, PerkinElmer Management Co., Shanghai, China). The temperature was heated from 30 °C to 600 °C at a rate of 10 °C/min.

### 2.9. Rheological Properties

Fresh extrudates were cut into cubes with side lengths of 20 mm and subjected to frequency sweep measurements using a rotational rheometer (HR20, TA Instruments, New Castle, DE, USA) equipped with 20 mm parallel plates. Frequency sweep tests were performed at a constant strain amplitude of 0.5% (within the linear viscoelastic region) over an angular frequency range of 0.1–100 rad/s. The storage modulus (G′) and loss modulus (G″) were recorded.

### 2.10. Texture Profile Analysis (TPA)

Texture profile analysis was performed using a texture analyzer (TMS-Pilot, Food Technology Corporation, Sterling, VA, USA) equipped with a 500 N load cell and a P50 cylindrical probe. The extrudates were cut into cubes with 2 cm sides and placed on the testing platform (75 mm diameter). According to the method described by Zheng et.al. [15] with minor modifications, the samples were compressed to 50% of the original height at a speed of 1 mm/s, with a 10 s interval between the two compression cycles. Textural parameters (e.g., hardness, springiness, chewiness and gumminess) were obtained using TL-Pro software (Version 1.8, Food Technology Corporation, Sterling, VA, USA).

### 2.11. Rehydration Properties

The dried extrudates were rehydrated in deionized water at 60 °C. Prior to immersion, the initial weight of the sample was recorded. Samples were independently rehydrated for 10, 20, 40, or 60 min and removed from the water at the corresponding time points. Excess surface moisture was removed using absorbent paper, and the rehydrated samples were immediately weighed. Separate samples were used for each rehydration period to ensure independent measurements. The moisture distribution and corresponding images of the rehydrated samples were analyzed using a low-field nuclear magnetic resonance analyzer (Niumag Co., Ltd., Shanghai, China). The rehydration ratio was determined using the following equation:

$$\mathrm{Rehydration}\;\mathrm{ratio}\;(\%)\;=\;\frac{W_{t}-W_{i}}{W_{i}}\;\times \;100$$

where *W_t_* represents the weight of the extrudate after rehydration for t min, and *W_i_* represents the initial weight of the dried extrudate.

### 2.12. Freeze–Thaw Stability Analysis

According to the methods described by Chen et.al. [16], the fresh extrudates were sliced into 1 cm × 1 cm × 1 cm cubes and frozen at −20 °C for 24 h. The frozen samples were then placed in cooking bags and thawed in a water bath (25 °C, 2 h). Upon thawing, the extrudates were gently dried with absorbent paper to remove surface moisture and subsequently weighed. This procedure was repeated for seven freeze–thaw cycles. The freeze–thaw loss was quantified as the percentage weight reduction following each cycle, calculated relative to the initial weight of the extrudates.

### 2.13. Electronic Tongue

The taste-related profiles of the samples were analyzed using an electronic-tongue system (SA-402B, Insent, Tokyo, Japan) equipped with lipid membrane-based potentiometric sensors for detecting taste-related chemical responses. The sensor array comprised five lipid membrane-based potentiometric sensors (CA0, C00, CT0, GL1, and AAE), corresponding to sourness, bitterness, saltiness, sweetness, and umami, respectively. For sample preparation, 30 g of the sample was homogenized in 150 mL of distilled water with a blender. Following centrifugation (6750× *g*, 10 min), the obtained supernatant was diluted 10-fold for measurement. The sensors were conditioned and calibrated before analysis. During each measurement cycle, the sensors were immersed sequentially in the reference solution and sample solution to record their membrane potentials. The relative sensor response was calculated as the potential difference between the sample and reference solutions (Vs-Vr), where Vs and Vr represent the respective membrane potentials. The sensors were cleaned between measurements to minimize carryover effects. Principal component analysis (PCA) was performed on the electronic-tongue sensor responses corresponding to sourness, bitterness, saltiness, sweetness, and umami to characterize differences in instrumental taste-related response patterns among treatments.

### 2.14. Electronic Nose

The volatile response profiles of the extrudates were characterized using an electronic-nose system (PEN3, Airsense Analytics GmbH, Schwerin, Germany). The instrument was equipped with an array of ten metal oxide semiconductor sensors (W1C, W5S, W3C, W6S, W5C, W1S, W1W, W2S, W2W, and W3S), exhibiting different response characteristics towards volatile compounds. Briefly, 1.0 g of the extrudates was transferred into a sealed headspace vial and equilibrated for 30 min to facilitate the enrichment of volatile organic compounds. During the measurement, the sensor cleaning and data acquisition periods were each set to 150 s, whereas the zeroing and sample preparation steps were set to 5 s. The sampling flow rate was maintained at 400 mL/min. Sensor responses were expressed as the ratio of sample gas conductivity to baseline conductivity (G/G_0_), where G and G_0_ represent the sensor conductance under sample exposure and reference conditions, respectively. Each treatment was measured in triplicate, and the sensor chamber was purged between measurements to restore the baseline. The sensor responses recorded between 120 and 130 s were averaged and processed using WinMuster software (version 1.6.2, Airsense Analytics GmbH, Schwerin, Germany).

### 2.15. Statistical Analysis

For each treatment, three independent extrusion runs were performed, with each run considered an experimental unit. Measurements obtained from the same run were treated as technical replicates and averaged before statistical analysis (*n* = 3). Data analysis was carried out with SPSS version 26.0, and differences between groups were assessed using one-way ANOVA followed by Duncan’s multiple range test (*p* < 0.05). For the rehydration and freeze–thaw experiments, a two-way repeated-measures ANOVA was performed to evaluate the effects of BSSP incorporation level, measurement time or freeze–thaw cycle. Graphs were created using OriginPro 2024 (OriginLab Corporation, Northampton, MA, USA) and GraphPad Prism (Version 10.5, GraphPad Software, Boston, MA, USA).

## 3. Results and Discussion

### 3.1. Extruder Responses

**Figure 2:**
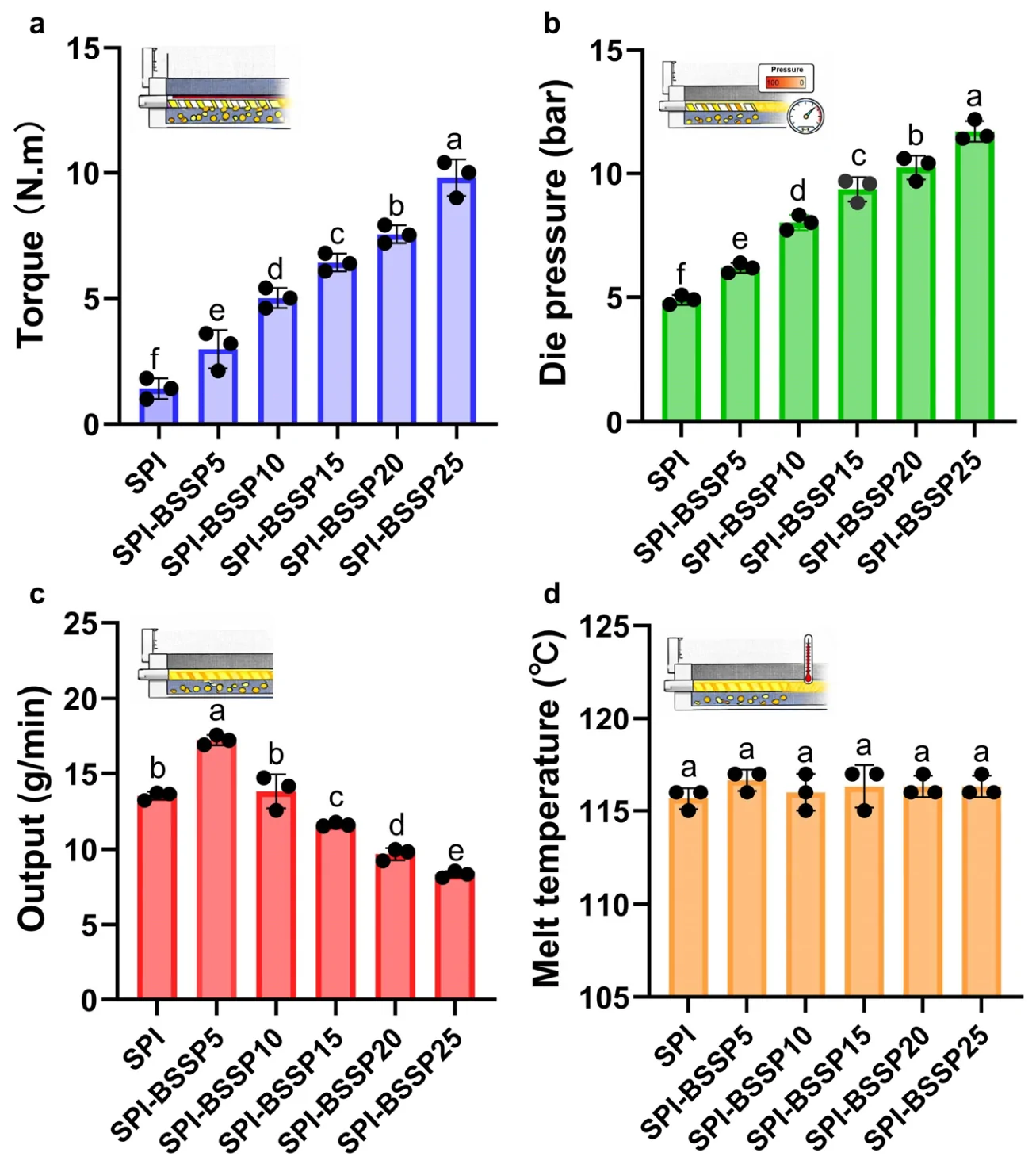
Response parameters of high-moisture extrusion system: (**a**) torque, (**b**) die pressure, (**c**) output, and (**d**) melt temperature. Bars represent mean values, error bars indicate standard deviations, and individual points correspond to three independent replicates (*n* = 3). Different lowercase letters (a–f) indicate significant differences among samples (*p* < 0.05). SPI represents high-moisture extrudates produced from a pure SPI matrix without BSSP addition. SPI-BSSP5, SPI-BSSP10, SPI-BSSP15, SPI-BSSP20, and SPI-BSSP25 denote extrudates produced from SPI/BSSP blends with mass ratios of 95/5, 90/10, 85/15, 80/20, and 75/25, respectively.

The system response parameters during high-moisture extrusion are presented in Figure 2. The incorporation of bamboo shoot shell powder (BSSP) significantly altered the mechanical response of the extrusion system. Torque increased progressively with increasing BSSP addition (Figure 2a), rising from 1.4 ± 0.4 N·m to 9.8 ± 0.7 N·m (*p* < 0.05). A comparable trend in die pressure (Figure 2b) was noted, suggesting increased melt viscosity and flow resistance in the barrel. A previous study has indicated that the introduction of dietary fiber during high-moisture extrusion tends to increase melt viscosity, which consequently results in higher mechanical energy and die pressure [17]. The rigid structure of the BSSP dietary fiber likely enhanced intermolecular friction and restricted molecular mobility within the protein matrix, thereby increasing mechanical energy input and pressure accumulation [18]. Extrusion output initially increased at 5% BSSP incorporation, followed by a progressive decrease at higher inclusion levels (Figure 2c). The reduction in output suggests hindered material transport and reduced conveying efficiency, consistent with the higher torque and pressure. Excessive fiber incorporation may disrupt melt continuity under constant operating conditions. Notably, the melt temperature showed no significant variation (Figure 2d), confirming stable thermal input. These findings suggested that increased torque and die pressure may be associated with changes in the viscosity of the protein matrix melt at appropriate BSSP incorporation levels.

### 3.2. Micromorphology Analysis

**Figure 3:**
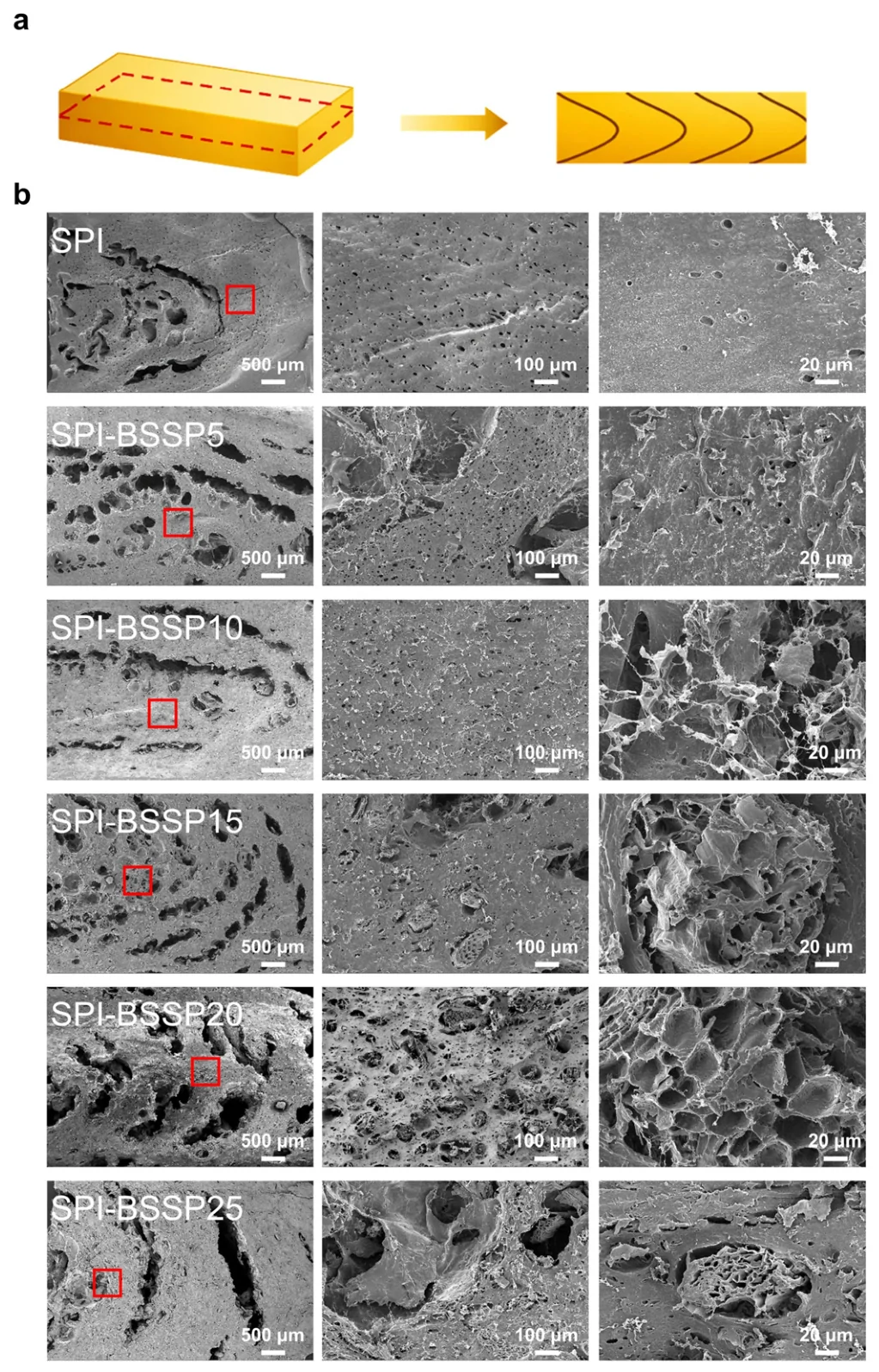
(**a**) Schematic diagram of the sectioning orientation for fresh extrudates. (**b**) Microstructural morphology (scale bars = 500 μm, 100 μm, and 20 μm) of high-moisture extrudates (the red box indicates the region selected for higher-magnification observation). SPI represents high-moisture extrudates produced from a pure SPI matrix without BSSP addition. SPI-BSSP5, SPI-BSSP10, SPI-BSSP15, SPI-BSSP20, and SPI-BSSP25 denote extrudates produced from SPI/BSSP blends with mass ratios of 95/5, 90/10, 85/15, 80/20, and 75/25, respectively.

Figure 3a illustrates the schematic diagram of sample sectioning along the extrusion direction for subsequent microstructural observation. As shown in Figure 3b, the SPI sample displayed a relatively compact matrix with irregular pores. With the incorporation of 5–10% BSSP, the microstructure evolved into channel-like pores aligned along the extrusion direction. This structural transition corresponded with the increased torque and die pressure, possibly reflecting changes in melt flow behavior during extrusion [19]. Similarly, previous studies have reported that sodium alginate incorporation altered the microstructure of soy protein extrudates, promoting the development of more organized and continuous textural structures [20]. When the BSSP level reached 15–20%, distinct parabolic structures emerged, accompanied by denser and more interconnected pore walls. Within this concentration range, BSSP may have acted as a reinforcing filler within the protein matrix, potentially promoting protein aggregation and stabilizing the network structure. In contrast, excessive addition (~25%) disrupted structural continuity, likely because insoluble fiber interfered with protein–protein interactions during melt solidification [21]. These results suggest that BSSP incorporation influences the pore morphology and microstructural organization of the extrudates.

### 3.3. FTIR Spectra Analysis

**Figure 4:**
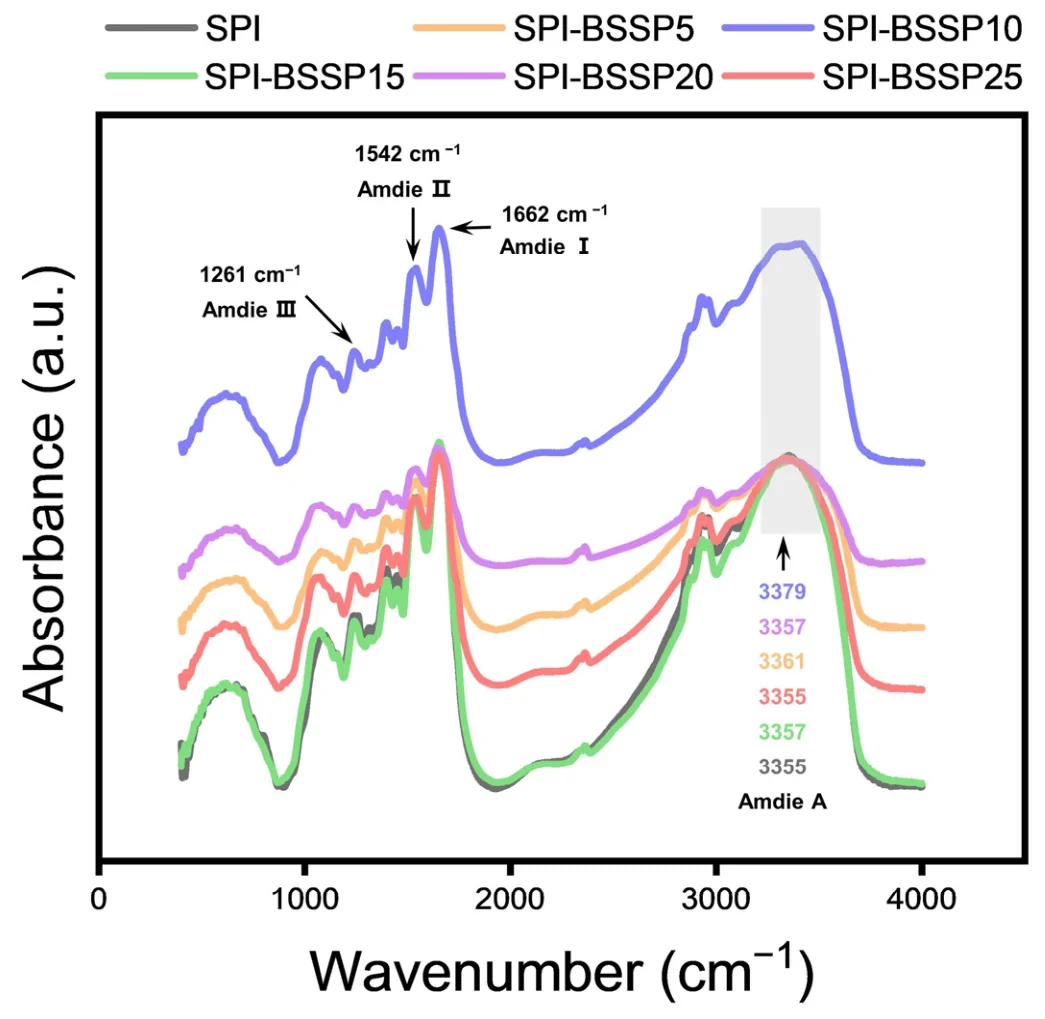
FTIR spectra of high-moisture extrudates. SPI represents high-moisture extrudates produced from a pure SPI matrix without BSSP addition. SPI-BSSP5, SPI-BSSP10, SPI-BSSP15, SPI-BSSP20, and SPI-BSSP25 denote extrudates produced from SPI/BSSP blends with mass ratios of 95/5, 90/10, 85/15, 80/20, and 75/25, respectively.

Figure 4 presents the FTIR spectra of high-moisture extrudates with different BSSP levels. All samples exhibited a broad Amide A band at 3355–3379 cm^−1^, attributed to the O–H stretching of hydroxyl groups and N–H stretching of peptide bonds [22]. Characteristic protein absorptions were observed at 1662 cm^−1^, 1524 cm^−1^, and 1261 cm^−1^, corresponding to peptide C=O stretching, C–N stretching, and N–H bending vibrations, respectively [23]. With increasing BSSP addition, the Amide I peak gradually shifted toward lower wavenumbers, suggesting enhanced intermolecular hydrogen bonding and stronger protein–fiber interactions during high-moisture extrusion. A red shift of the Amide I band has been reported in polysaccharide–protein extrusion systems, where the incorporation of non-starch polysaccharides strengthened hydrogen bonding interactions [24]. To quantify the relative proportions of protein secondary structures, the amide I region (1600–1700 cm^−1^) was analyzed by peak deconvolution and curve fitting using PeakFit software (Table 1). Moderate BSSP incorporation (15–20%) increased β-sheet and β-turn contents while reducing α-helix and random coil fractions, indicating a transition from disordered to ordered conformations. The presence of β-sheet structures suggests enhanced protein reassociation during extrusion, which contributes to improved structural stability of extrudates [25]. However, excessive BSSP (~25%) reduced β-sheet content, likely because high levels of insoluble fiber hinder protein–protein interactions and disrupt network formation. These findings suggested that appropriate BSSP incorporation altered protein secondary structure, as reflected by increased β-sheet and β-turn proportions in high-moisture extrudates.

### 3.4. ThT Fluorescence

**Figure 5:**
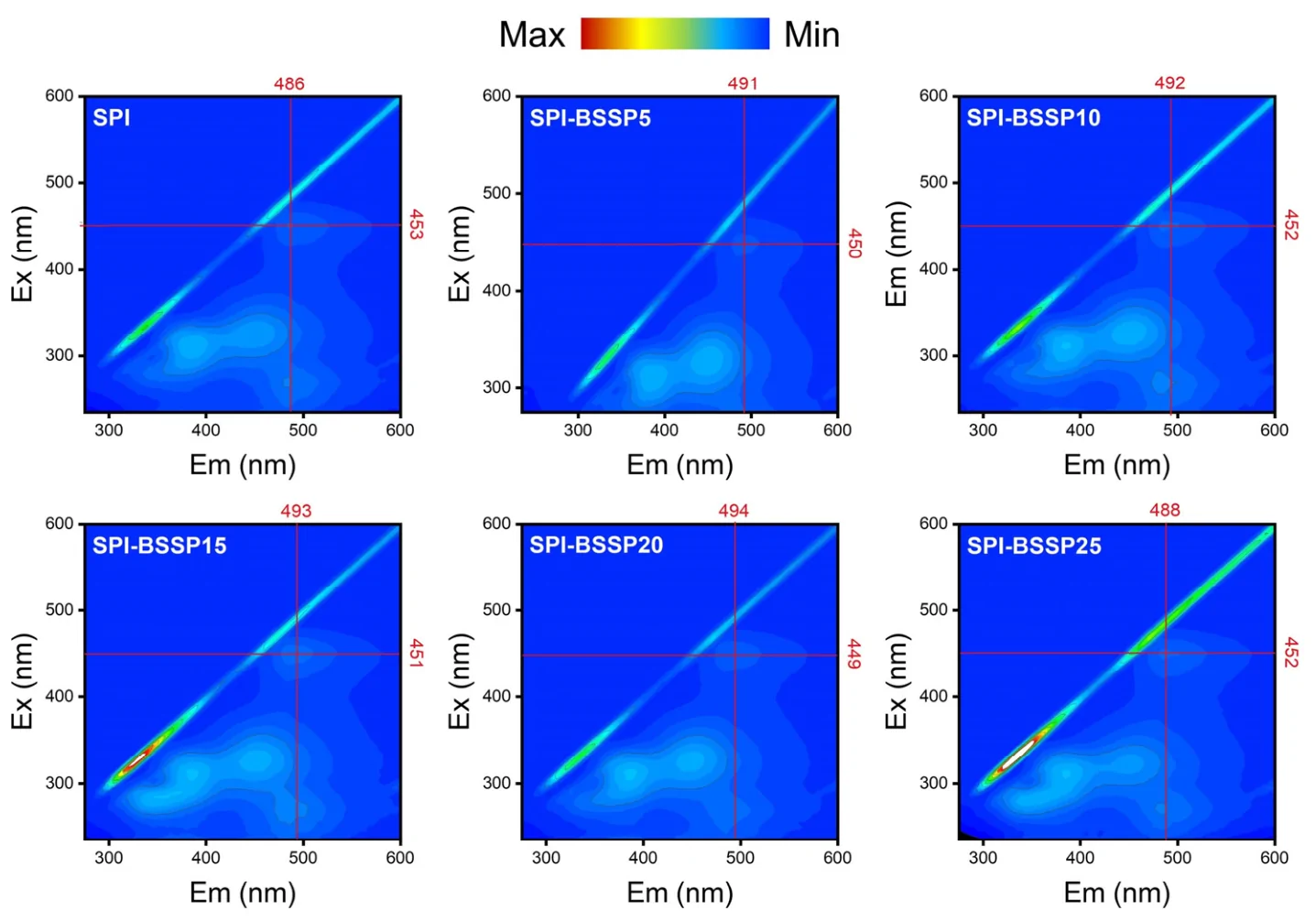
Three-dimensional fluorescence spectra of high-moisture extrudates. SPI represents high-moisture extrudates produced from a pure SPI matrix without BSSP addition. SPI-BSSP5, SPI-BSSP10, SPI-BSSP15, SPI-BSSP20, and SPI-BSSP25 denote extrudates produced from SPI/BSSP blends with mass ratios of 95/5, 90/10, 85/15, 80/20, and 75/25, respectively.

ThT fluorescence is widely used to probe β-sheet-rich protein aggregates. With increasing BSSP addition (0~15%), the extrudates of fluorescence intensity progressively increased, accompanied by a slight red shift of the emission maximum from 486 to 493 nm (Figure 5). This trend suggests that BSSP incorporation may promote development of ordered β-sheet assemblies [26]. Such structural ordering likely arises from the cooperative shear experienced by protein chains mediated by dietary fibers during extrusion, which enhances molecular alignment [27]. At a higher BSSP level (~25%), fluorescence intensity declined, suggesting a reduction in ThT binding sites or changes in the aggregation characteristics of the protein matrix. An inhibitory effect has been reported in protein–polysaccharide systems, where excessive polysaccharide disrupted protein–protein interactions and limited the formation of ordered β-sheet networks during aggregation [28]. These results indicated that BSSP incorporation levels (15~20%) facilitated the assembly of ordered β-sheet aggregates during high-moisture extrusion, offering new insights into the structural design of meat analogues.

### 3.5. Intermolecular Interaction Forces

To determine the primary intermolecular forces responsible for protein stability, intermolecular forces in the extrudates were quantitatively analyzed (Table 2). Relative to the SPI sample, the SPI-BSSP5 sample showed an approximately 2.9-fold increase in hydrogen bonding, alongside a ~45% reduction in hydrophobic interactions. This shift suggests that hydroxyl groups from dietary fiber formed competitive hydrogen bonds with polar protein side chains, thereby weakening protein–protein hydrophobic association. During thermomechanical processing, insoluble dietary fibers may competitively bind water molecules, reducing protein hydration and facilitating the exposure of hydrophobic groups, which in turn enhances protein–protein interactions [29]. As the BSSP addition approached 15%, disulfide bond content reached a maximum in SPI-BSSP15 (4.28 ± 0.34 mg/mL), markedly higher than in the SPI sample (1.89 ± 0.26 mg/mL, *p* < 0.05). This result suggests that dietary fiber-induced thermo-mechanical effects promote disulfide bond reshuffling and strengthen covalent cross-linking in the protein matrix [30]. Notably, both hydrophobic interactions and disulfide bonds declined at 25% BSSP addition, consistent with the reduced ThT fluorescence. These results demonstrated that BSSP enhanced both covalent cross-linking and non-covalent interactions among protein components, thereby contributing to a more resilient protein matrix.

### 3.6. Thermal Properties

**Figure 6:**
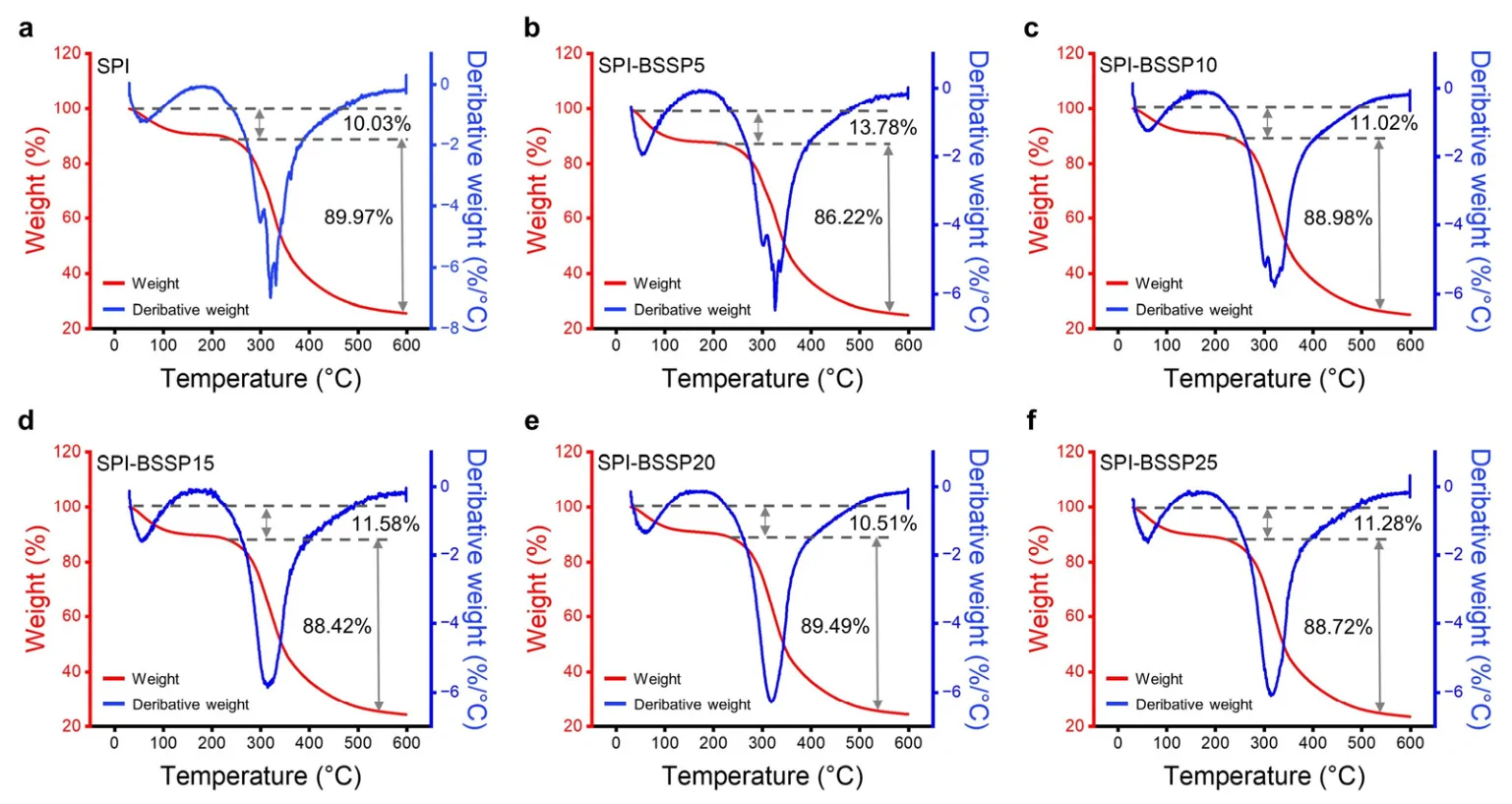
Thermogravimetric (TGA, red) and derivative thermogravimetric (DTG, blue) curves of (**a**) SPI, (**b**) SPI-BSSP5, (**c**) SPI-BSSP10, (**d**) SPI-BSSP15, (**e**) SPI-BSSP20, and (**f**) SPI-BSSP25. SPI represents high-moisture extrudates produced from a pure SPI matrix without BSSP addition. SPI-BSSP5, SPI-BSSP10, SPI-BSSP15, SPI-BSSP20, and SPI-BSSP25 denote extrudates produced from SPI/BSSP blends with mass ratios of 95/5, 90/10, 85/15, 80/20, and 75/25, respectively.

Thermal stability of the high-moisture extrudates was evaluated using TGA and DTG analyses. As shown in Figure 6, all samples displayed a typical two-step mass-loss profile. In the initial stage (30–150 °C), both free water and bound water are evaporated. Compared with the SPI sample, the SPI-BSSP5 sample showed a 7.48% higher weight loss in this stage, suggesting that BSSP incorporation enhanced water retention with the extrudates. This phenomenon may be due to the oriented fibrous structures and multiscale pore channels formed during extrusion, which facilitate the retention of bound water in the protein matrix [31]. The primary degradation zone (200–380 °C) corresponds to protein backbone cleavage accompanied by side-chain scission [32]. Upon BSSP addition, the DTG peak of the extrudate samples shifted to higher temperatures, indicating improved thermal stability (Figure 6). This finding is consistent with the report by Wang et al. [33], indicating that the polysaccharides reinforce intermolecular networks, thereby retarding the thermal degradation of the protein matrix. At the molecular level, coupled shear and thermal energy may influence sulfhydryl-disulfide exchange and hydrophobic interactions, potentially contributing to changes in protein matrix organization [34]. These findings demonstrated that high-moisture extrusion significantly enhanced thermal stability by strengthening fiber–protein interactions and establishing an oriented network.

### 3.7. Rheological Properties

**Figure 7:**
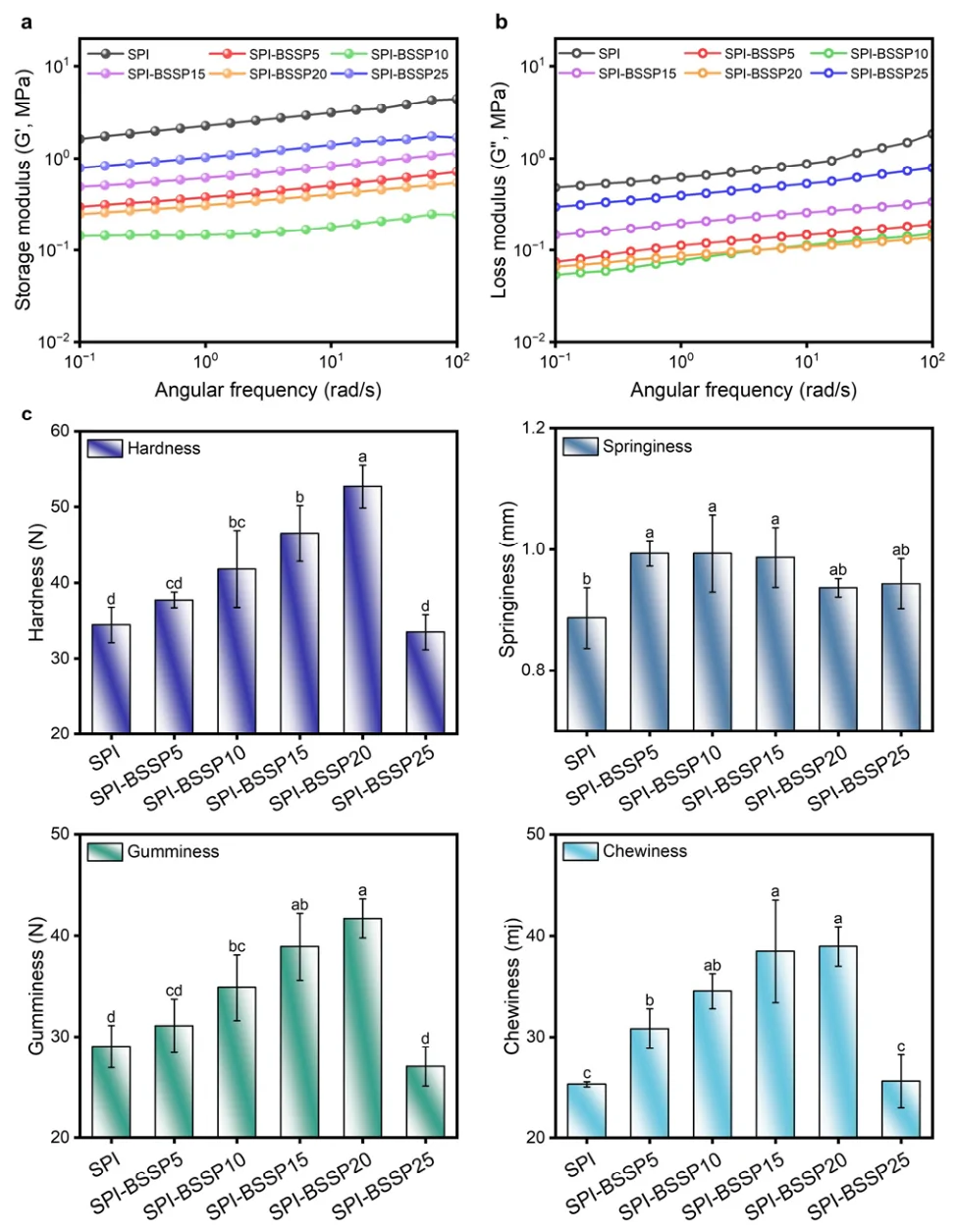
(**a**) Storage modulus (G′, solid circle) and (**b**) loss modulus (G″, hollow circle) as a function of frequency. (**c**) Textural properties (e.g., hardness, springiness, gumminess and chewiness) of high-moisture extrudates. SPI represents high-moisture extrudates produced from a pure SPI matrix without BSSP addition. SPI-BSSP5, SPI-BSSP10, SPI-BSSP15, SPI-BSSP20, and SPI-BSSP25 denote extrudates produced from SPI/BSSP blends with mass ratios of 95/5, 90/10, 85/15, 80/20, and 75/25, respectively. Different lowercase letters indicate significant differences among samples (*p* < 0.05).

Figure 7a,b depict the frequency-dependent viscoelastic profiles of high-moisture extrudates with varying levels of BSSP incorporated. For all samples, the storage modulus (G′) exceeded the loss modulus (G″), with both increasing progressively with rising frequency. This response is attributed to the extrusion-induced ordering and assembly of protein into a highly organized three-dimensional network [35]. Notably, the SPI sample showed the highest G′ and G″ values, suggesting a stronger viscoelastic response of the protein network in the absence of BSSP. At a low addition level (~10%), both G′ and G″ values decreased markedly. This reduction may be attributed to the disruption of the continuous protein network by BSSP, together with changes in water distribution and protein–protein interactions, thereby weakening the viscoelastic response of the protein matrix. When the BSSP content was increased to higher levels (≥20%), the moduli increased again. This suggests that the dietary fiber participates in forming a protein–fiber composite network. The resulting structural reinforcement enhances stress-transfer efficiency and elastic recovery, leading to higher stiffness and improved structural stability of the extrudates [36]. Precise control of BSSP incorporation levels allowed regulation of the viscoelastic behavior of high-moisture extruded soy protein systems.

### 3.8. Textural Properties Analysis

Figure 7c illustrates the influence of different BSSP addition levels on the textural properties of the extrudates, including hardness, springiness, gumminess, and chewiness. As the BSSP content increased from 0% to 20%, hardness, gumminess, and chewiness rose significantly (*p* < 0.05). This enhancement is primarily attributed to BSSP acting as a rigid filler embedded within the protein matrix, which enhances mechanical strength through improved interfacial interactions [37]. Similar reinforcement effects of dietary fibers on protein networks have been widely reported in a previous study [38]. Under high-shear conditions, the dietary fibers aligned along the melt-flow direction and induced protein crosslinking on their surfaces, forming a compression-resistant network [39]. Conversely, springiness remained relatively stable at high levels, suggesting that elastic recovery was governed primarily by the protein network, with limited influence from BSSP addition. However, exceeding 20% BSSP led to a decline in hardness and chewiness, indicating that excessive fiber disrupts structural integrity. These findings indicated that incorporating BSSP was critical for enhancing textural properties, providing a basis for designing high-quality plant-based meat analogues.

### 3.9. Rehydration Stability

**Figure 8:**
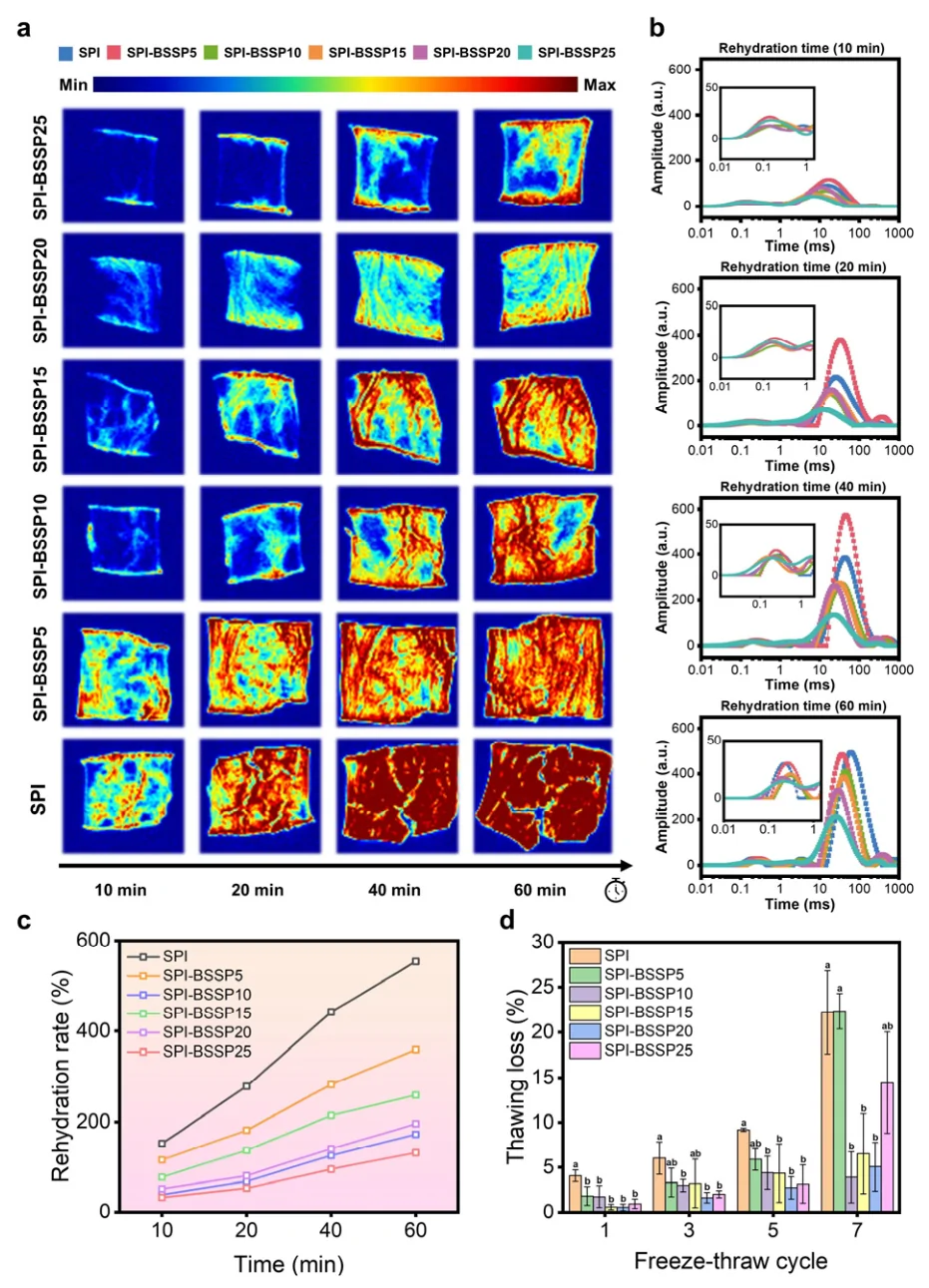
(**a**) Low-field resonance nuclear magnetic resonance (LF-NMR) images. (**b**) T2 relaxation time distribution curves. (**c**) Rehydration ratio of the high-moisture extrudates as a function of rehydration time (10, 20, 40, and 60 min). (**d**) Thawing loss of extrudate samples after different freeze–thaw cycles. Different lowercase letters indicate significant differences among samples (*p* < 0.05). SPI represents high-moisture extrudates produced from a pure SPI matrix without BSSP addition. SPI-BSSP5, SPI-BSSP10, SPI-BSSP15, SPI-BSSP20, and SPI-BSSP25 denote extrudates produced from SPI/BSSP blends with mass ratios of 95/5, 90/10, 85/15, 80/20, and 75/25, respectively.

Figure 8a depicted the magnetic resonance imaging (MRI) of the samples during rehydration at 60 °C. All samples exhibited a progressive water penetration pattern from the surface towards the interior, with image brightness increasing over time, reflecting a rise in proton density. The SPI sample showed relatively higher signal intensity and developed a uniform water distribution. Meanwhile, the reduction in signal intensity with increasing BSSP concentration suggests that BSSP helped preserve network integrity and modulated water migration pathways. This trend was supported by the LF-NMR results (Figure 8b) and the rehydration rates (Figure 8c). After 60 min of rehydration, the SPI sample appeared to have the highest peak amplitude and a rehydration rate of 553.58 ± 1.07%, compared with 131.79 ± 0.26% for the SPI-BSSP25 sample. The lower rehydration ratio of BSSP-containing extrudates may be associated with differences in their pore morphology and water-holding capacity, which influence water penetration and retention during hydration [17]. Previous studies have reported that polysaccharides could modify protein network structures through intermolecular interactions, thereby affecting water distribution and hydration behavior [33]. Similarly, dietary fiber derived from BSSP facilitates the formation of a denser cross-linked matrix, thereby restricting free water diffusion. These findings indicated that the incorporation of BSSP effectively regulated water migration and improved rehydration stability.

### 3.10. Freeze–Thaw Stability

As illustrated in Figure 8d, the thawing loss of extrudates increased significantly with cumulative freeze–thaw cycles, reflecting freeze-induced water loss and the dynamic evolution of the protein network structures. In the first five cycles, BSSP-incorporated extrudates exhibited higher water holding capacity compared to the SPI sample. Notably, the SPI-BSSP20 sample maintained a low thawing loss (~3%), whereas the SPI sample rose to ~10% (*p* < 0.05). This indicates that the dietary fiber–protein interactions enhance structural organization and protect against mechanical rupture caused by ice crystal growth [40]. A slight increase in thawing loss was observed in BSSP-containing extrudates during the early cycles, which may be related to the formation of a denser composite network that limited water mobility. These observations agree with the report by Zheng et al. [41], who argued that the incorporation of fiber-rich structure materials could effectively regulate the water distribution within the protein matrix. Following seven cycles, the SPI sample exhibited a sharp rise in thawing loss (~20%, *p* < 0.05), indicating that the repeated freeze–thaw had exceeded the tolerance of its structure. In contrast, the SPI-BSSP10 sample maintained a significantly lower thawing loss of 3.90 ± 2.85% than the SPI sample (22.23 ± 4.60%, *p* < 0.05). These results demonstrated that incorporating BSSP was beneficial to create a robust gel network that maintained structural integrity and improved freeze–thaw resistance during rehydration.

### 3.11. Electronic-Tongue and Electronic-Nose Analyses

**Figure 9:**
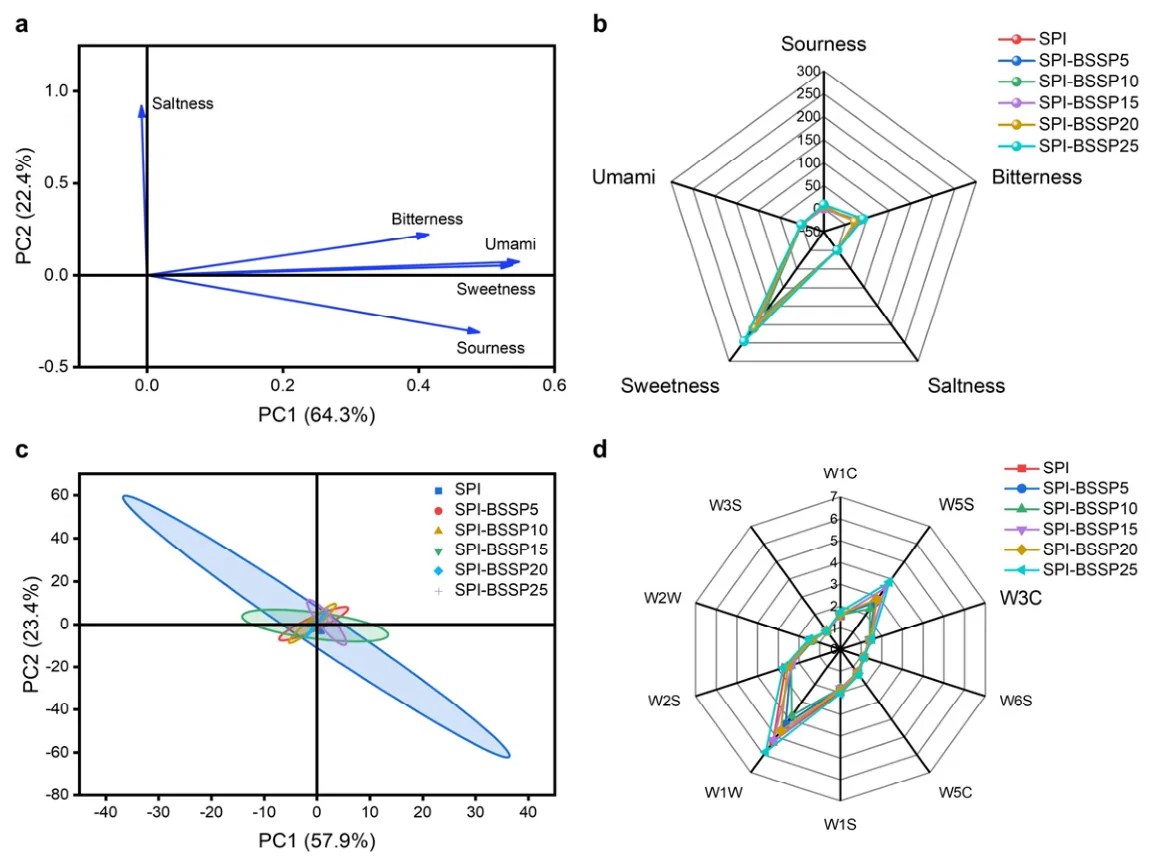
(**a**) Principal component analysis of electronic-tongue responses. (**b**) Electronic-tongue radar diagram. (**c**) Electronic-nose PCA analysis diagram. (**d**) Electronic-nose radar diagram. SPI represents high-moisture extrudates produced from a pure SPI matrix without BSSP addition. SPI-BSSP5, SPI-BSSP10, SPI-BSSP15, SPI-BSSP20, and SPI-BSSP25 denote extrudates produced from SPI/BSSP blends with mass ratios of 95/5, 90/10, 85/15, 80/20, and 75/25, respectively.

The taste-related and volatile response profiles of the high-moisture extrudates were characterized using an electronic tongue and an electronic nose, as shown in Figure 9. Principal component analysis of the E-tongue (PC1: 64.3%) captured distinct taste differentiation among samples. Specifically, BSSP incorporation increased the responses from the sweetness- and umami-related sensors while decreasing those from the bitterness- and sourness-related sensors (Figure 9b). This effect may be associated with the adsorption and masking ability of dietary fibers toward bitter peptides and acidic compounds generated during high-moisture extrusion [42]. Regarding volatile-response profiles, Figure 9c showed that PC1 and PC2 accounted for 57.9% and 23.4%, respectively. The electronic-nose responses were primarily characterized by signals from W1W, W1S, and W2S. Notably, BSSP-containing extrudates exhibited stronger responses from these sensors, indicating differences in the volatile-response patterns among the extrudates (Figure 9d). Such variations may be associated with differences in volatile release or retention within the protein–fiber matrix [43]. These findings indicated that BSSP incorporation altered the electronic-tongue and electronic-nose response profiles of high-moisture extrudates.

## 4. Conclusions

In this work, the incorporation of BSSP during high-moisture extrusion significantly increased screw torque and die pressure. Compared with the SPI sample, the BSSP-containing extrudates exhibited more pronounced line-like (cracks) microstructures and improved thermal stability. ThT fluorescence analysis suggested changes in the formation of β-sheet-rich protein aggregates following BSSP incorporation. Structural analyses showed that moderate BSSP addition increased β-sheet and β-turn contents while decreasing α-helix and random coil fractions. This structural transition may be associated with changes in hydrogen bonding and hydrophobic interactions within the protein matrix. Additionally, appropriate BSSP incorporation improved the freeze–thaw stability and textural properties of the extrudates. Based on these observations, this study provided valuable insights into the role of bamboo shoot shell powder in regulating the structural and textural properties of plant-based meat analogues, offering a potential approach for developing high-quality meat analogues. Future research should further investigate the effects of different dietary fiber types and incorporation levels on fibrous structure development during high-moisture extrusion of plant proteins.

## Figures and Tables

**Table 1:** Secondary structure proportion of high-moisture extrudates.

Samples	β-Sheet (%)	β-Turn (%)	α-Helix (%)	Random Coil (%)
SPI	19.06 ± 1.09 ^bc^	16.90 ± 1.01 ^c^	30.00 ± 1.23 ^a^	34.04 ± 0.87 ^ab^
SPI-BSSP5	17.81 ± 1.41 ^c^	16.34 ± 1.17 ^c^	30.82 ± 1.53 ^a^	35.03 ± 1.05 ^a^
SPI-BSSP10	17.95 ± 1.19 ^bc^	17.72 ± 1.13 ^bc^	30.73 ± 2.40 ^a^	33.60 ± 0.08 ^bc^
SPI-BSSP15	19.25 ± 0.01 ^bc^	18.64 ± 1.13 ^ab^	29.46 ± 0.04 ^ab^	32.64 ± 0.10 ^cd^
SPI-BSSP20	21.76 ± 0.02 ^a^	18.42 ±0.09 ^ab^	27.48 ± 0.01 ^b^	32.35 ± 0.08 ^d^
SPI-BSSP25	19.54 ± 0.00 ^b^	19.41 ± 0.09 ^a^	28.50 ± 0.00 ^ab^	32.56 ± 0.09 ^cd^

Note: Different letters indicate significant differences among treatments (*p* < 0.05).

**Table 2:** The interaction force of high-moisture extrudates.

Samples	Ionic Bonds (mg/mL)	Hydrogen Bonds (mg/mL)	Hydrophobic Interactions (mg/mL)	Disulfide Bonds (mg/mL)
SPI	0.60 ± 0.01 ^ab^	0.61 ± 0.05 ^c^	3.74 ± 0.29 ^a^	1.89 ± 0.26 ^c^
SPI-BSSP5	0.39 ± 0.08 ^ab^	1.75 ± 0.11 ^a^	2.04 ± 0.19 ^c^	2.40 ± 0.37 ^bc^
SPI-BSSP10	0.53 ± 0.19 ^ab^	0.18 ± 0.18 ^d^	1.82 ± 0.28 ^c^	3.88 ±0.19 ^a^
SPI-BSSP15	0.92 ± 0.10 ^a^	1.40 ± 0.12 ^b^	3.43 ± 0.09 ^a^	4.28 ± 0.34 ^a^
SPI-BSSP20	0.24 ± 0.14 ^b^	0.65 ± 0.04 ^c^	2.65 ± 0.11 ^b^	2.88 ± 0.66 ^b^
SPI-BSSP25	0.46 ± 0.08 ^ab^	0.51 ± 0.03 ^c^	1.40 ± 0.06 ^d^	1.21 ± 0.15 ^d^

Note: Different letters indicate significant differences among treatments (*p* < 0.05).

## Data Availability

The original contributions presented in this study are included in the article. The raw data supporting the findings of this study are available from the corresponding authors upon reasonable request.

## Abbreviations

The following abbreviations are used in this manuscript:

TVPs	Textured vegetable proteins
HME	High-moisture extrusion
BSSP	Bamboo shoot shell powder
SPI	Soy protein isolate
ThT	Thioflavin
LF-NMR	Low-field resonance nuclear magnetic resonance
